# Safety and efficacy of endoscopic cyanoacrylate injection in the management of gastric varices: A systematic review and meta‐analysis

**DOI:** 10.1002/jgh3.12629

**Published:** 2021-07-30

**Authors:** Sakkarin Chirapongsathorn, Wuttiporn Manatsathit, Ann Farrell, Anuchit Suksamai

**Affiliations:** ^1^ Division of Gastroenterology and Hepatology, Department of Medicine Phramongkutklao Hospital, College of Medicine, Royal Thai Army Bangkok Thailand; ^2^ Department of Gastroenterology and Hepatology University of Nebraska Medical Center Omaha Nebraska USA; ^3^ Mayo Clinic Libraries Mayo Clinic Rochester Minnesota USA

**Keywords:** gastric varices, glue injection, hepatology, portal hypertension

## Abstract

**Background and Aim:**

Bleeding from gastric varices is a catastrophic event and poses difficulty in management. The efficacy and safety of cyanoacrylate injection remain unclear. We performed a systematic review and meta‐analysis to evaluate the effect of endoscopic cyanoacrylate injection in the management of gastric varices.

**Methods:**

We conducted a comprehensive search of MEDLINE, Embase, Web of Science, Scopus databases, and Cochrane Database of Systematic Reviews through November 2020 and manually reviewed the literature. Trial‐specific risk ratios (RRs) were estimated and pooled using random‐effect model meta‐analysis.

**Results:**

We included seven randomized controlled trials (six for secondary prophylaxis and one for primary prophylaxis) at low risk of bias in which 126 deaths were reported among 583 patients with gastric varices. All studies reported the use of N‐butyl‐2‐cyanoacrylate glue. Cyanoacrylate use was associated with significantly lower all‐cause mortality (RR, 0.59; 95% confidence interval [CI], 0.36–0.98; I^2^ = 41%) and rebleeding rate after hemostasis (RR, 0.49; 95% CI, 0.35–0.68, I^2^ = 0%) compared with any other treatment approach not involving cyanoacrylate. When cyanoacrylate was compared with each individual treatment approach (propranolol only, band ligation, sclerotherapy with alcohol or ethanolamine), data comprised sparse limited comparative conclusions. The use of cyanoacrylate injection was not associated with an increase in serious adverse events. The quality of evidence is moderate, graded down due to the small number of events and wide CIs.

**Conclusion:**

The use of endoscopic cyanoacrylate injection therapy for gastric varices may be associated with lower all‐cause mortality and better hemostasis compared with other therapies.

## Graphical abstract

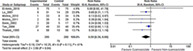

## Introduction

Gastric varices (GV) are a less common cause of upper gastrointestinal bleeding among patients with cirrhosis; however, it poses significantly higher morbidity and mortality compared with esophageal varices (EV).^1^ Unlike management of EV bleeding, management of GV widely varies depending on type of GV, experience of each endoscopist and endoscopic center, and availability of treatment option in each institute.

Fundamentally, GV can be classified into gastroesophageal varices (GOV) and isolated gastric varices (IGV) depending on the presence or absence of extended EV.^1^ GOV is further classified as GOV1, which is an extension of EV into the gastric cardia, and GOV2, which is an extension of EV into the fundus. IGV is classified as IGV1, which is found in the fundus and IGV2, which presents in other locations.^1^ Treatment of GOV1 is similar to treating of EV while treating of GOV2 and IGV1 is more challenging due to the location and difficulty in accessing upon the forward view of the lesion. Treatment options of GOV2, IGV1, and persistent GOV1 after obliterating the EV include injecting sclerosing agents, transjugular intrahepatic portosystemic shunt (TIPS), and balloon‐occluded retrograde transvenous obliteration of gastric varices (BRTO).^2^ Outside North America, endoscopic cyanoacrylate injection is considered the treatment of choice to obliterate these varices while BRTO and TIPS are more commonly used in Japan and the United States, respectively.^3^, ^4^, ^5^ Although growing anecdotal evidence from retrospective and non‐randomized control trial studies suggests that cyanoacrylate is effective, injecting sclerosing agents is still not widely available or universally used in the United States.

Due to disparity in managing GV and unclear efficacy of cyanoacrylate compared with other treatment modalities, we conducted a systematic review and meta‐analysis comparing outcomes of patients with cirrhosis undergoing cyanoacrylate injection and other treatment options including banding, other sclerosing agents, and beta‐blocker alone.

## Methods

This meta‐analysis was reported according to the Preferred Reporting Items for Systematic Reviews and Meta‐Analyses statement.^6^ The process followed an a priori established protocol. This study was exempt from institutional review board approval because the analysis involved only de‐identified data. All individual studies had received local institutional review board approval.

### Search strategy

A systematic literature search of Ovid MEDLINE (1946 through 13 November 2020), Ovid Embase (1988 through 13 November 2020), Web of Science (1993 through 13 November 2020), and Scopus databases was conducted by an experienced medical librarian and informatics specialist (Ann Farrell) for all relevant articles on the effect of endoscopic cyanoacrylate injection and the outcomes among patients with GV. Terms used in the search included MEDLINE and Embase subject headings as well as keywords (which are shown in Appendix A). Two investigators (Sakkarin Chirapongsathorn and Wuttiporn Manatsathit) independently reviewed the titles and abstracts of the studies identified in the search based on prespecified inclusion and exclusion criteria. The full text of the included studies from this first phase then was reviewed independently to determine whether or not they met the inclusion criteria. Next, we manually searched the bibliographies of the selected articles as well as review articles on this topic to identify additional relevant references. We also performed a manual search of conference proceedings from major gastroenterology and hepatology meetings (The Liver Meeting hosted by The American Association for the Study of Liver Diseases, The International Liver Congress hosted by The European Association for the Study of the Liver, and Digestive Disease Week hosted by The American Gastroenterological Association, from 2010 to 2020) for additional abstracts on the topic. When additional information was needed, we contacted the corresponding investigators of eligible studies using e‐mail, including a cover letter detailing the objective of this meta‐analysis, background information, and a Microsoft Excel (Redmond, WA, USA) document containing the required data collection form. When investigators did not respond, we sent another reminder e‐mail 2–4 weeks after the first one. When no response was received to the second e‐mail and the published data were unable to be analyzed, the study was excluded.

### Selection criteria

We included all randomized controlled trials (RCTs) meeting the following inclusion criteria: (i) patients with established GV, (ii) evaluated and clearly defined exposure to endoscopic cyanoacrylate injection, (iii) reported survival or mortality of subjects receiving endoscopic cyanoacrylate injection, and (iv) reported relative risks (RRs), odds ratios, or data provided for their calculation. Inclusion was not otherwise restricted by study size, language, or publication type. We excluded studies in which subjects presented unclearly identified GV and observational studies including case series and case reports.

When multiple publications used the same cohort, only data from the most recent and more comprehensive report were included. The flow diagram in Appendix C summarizes the study identification and selection process.

### Data abstraction

Data were abstracted independently in a standardized form by two reviewers (Sakkarin Chirapongsathorn and Wuttiporn Manatsathit). The following data were collected from each study: study design, time period of study/year of publication country of the sampled populations, primary outcome reported, types of medication, dose and duration of cyanoacrylate use, type of control interventions, total number of individuals in each group (exposed *vs* unexposed), and RRs with their 95% confidence intervals (CIs). Data on the following patient characteristics were extracted: age, sex, Child–Pugh class,^7^ model for end‐stage liver disease score,^8^ etiology of cirrhosis, and number of patients defined as having refractory ascites. Disagreements were resolved by consensus among investigators. Patients with GV were defined as having bleeding GV requiring treatment. When data were available only in figures, (i.e. survival curves), estimation of a 2 × 2 contingency table was performed independently by two reviewers (Sakkarin Chirapongsathorn and Wuttiporn Manatsathit) using the reported sample size from the text (number of patients at risk) and an effect size estimate from figures. Consensus was reached on such estimates among reviewers.

### Quality assessment

The risk of bias and methodologic quality of included studies were assessed by two authors independently (Sakkarin Chirapongsathorn and Wuttiporn Manatsathit) using the Cochrane risk of bias tool for RCTs. Disagreements regarding risk of bias assessment were resolved by consensus among investigators (Sakkarin Chirapongsathorn, Wuttiporn Manatsathit, and Anuchit Suksamai). The corresponding investigator was contacted when relevant information was unreported. Details of the Cochrane risk of bias tool for RCTs are summarized in Appendix B.^9^

### Outcomes

The primary analysis focused on assessing mortality among patients with GV using endoscopic cyanoacrylate injection. Anticipated hypotheses to explain potential heterogeneity in the effect included study design (RCTs), location of study, and type and dose of endoscopic cyanoacrylate injection. The secondary analysis focused on assessing hemostasis control among patients with bleeding GV using endoscopic cyanoacrylate injection.

### Statistical analysis

Because we expected heterogeneity across studies, all analyses were performed using a random‐effect model to pool RRs and 95% CIs.^10^ Heterogeneity was assessed using the I^2^ statistic. This statistic represents the proportion of variability that is unattributable to chance. I^2^ values greater than 50% indicate substantial heterogeneity. We assessed the potential for publication bias quantitatively using the Begg and Mazumdar^11^ adjusted rank correlation test, and qualitatively by funnel plots. All *P* values were two‐sided, and a *P* value less than 0.05 was considered statistically significant. All calculations and graphs were produced using Review Manager (RevMan) Version 5.3 (The Nordic Cochrane Centre, Copenhagen, Denmark) and the Begg adjusted rank correlation test was performed using comprehensive meta‐analysis.^12^

## Results

### Search results

Of the 1107 unique studies identified using our search criteria, a total of 10 studies met the search criteria. Ten randomized controlled studies were initially included and three studies were subsequently excluded due to absence of inclusion criteria in two studies and mixed population of EV and GV in another study, finally a total of seven studies fulfilled our inclusion criteria and were included in the meta‐analysis.^13^, ^14^, ^15^, ^16^, ^17^, ^18^, ^19^ Process details are provided in Appendix A. We found that one of the studies included was published only as an abstract^13^ and two of the studies included had enrolled patients with cirrhosis and non‐cirrhotic portal hypertension.^13^, ^19^ One of the studies included had enrolled patients with cirrhosis and GV who never bled and considered endoscopic cyanoacrylate injection as primary prophylaxis for bleeding. The included studies collectively reported 126 deaths among 583 patients with GV. All studies reported the use of N‐butyl‐2‐cyanoacrylate glue. Among those who died, 50 (40%) subjects received endoscopic cyanoacrylate injection and 76 (60%) subjects received other interventions, typically endoscopic band ligation, endoscopic ethanolamine injection, endoscopic alcohol injection, and oral propranolol treatment.

### Characteristics of included studies

The characteristics of the included studies are summarized in Table 1 and Tables S1 and S2. All studies were published in peer‐review journals. Comparison treatment interventions for GV were band ligating in three studies, propranolol in two studies, ethanolamine oleate in one study, and alcohol in one study. Primary prophylaxis for GV bleeding was primary endpoint in one study while secondary prophylaxis was primary endpoint in the other six studies. Three studies enrolled patients with GOV2 and IGV1^14^, ^17^, ^19^ while another three studies enrolled patients with GOV1, GOV2, and IGV1.^15^, ^16^, ^18^ A study by El Amin *et al*. only included patients with GOV1. Regarding etiology of portal hypertension, two studies^13^, ^19^ included all types of portal hypertension, whereas the rest of the studies only included patients with cirrhosis. Follow‐up duration varied from 6 months up to more than 2 years. Six studies were conducted in Asia (three studies in India, two studies in Taiwan, and one study from Egypt) and one study from Spain. Most studies reported secondary prophylaxis role of endoscopic cyanoacrylate injection except Mishra *et al*.^14^ Most studies reported cirrhosis related to alcoholic liver disease and viral hepatitis as the leading etiology.

**Table 1:** Characteristic of studies

			Characteristic of studied patients		
Studies	Primary endpoint	Comparison	Type of GOV (*n*)	Etiology of PTN	Duration of follow up
Mishra (2011)	Primary prophylaxis	Propranolol	GOV2 (51) IGV1 (8)	Cirrhosis	26 months (3–34)
Lo (2001)	Secondary prophylaxis	Band ligation	GOV1 (37) GOV2 (13) IGV1 (5)	Cirrhosis	14 months (GVO) 9 months (GVL)
Tan (2006)	Secondary prophylaxis	Band ligation	GOV1 (53) GOV2 (25) IGV1 (19)	Cirrhosis	610 ± 603.04 days (GVL) 680.67 ± 710.54 days (GVO)
El Amin (2010)	Secondary prophylaxis	Band ligation	GOV1 (150)	Cirrhosis and non‐cirrhotic	6 months
Mishra (2010)	Secondary prophylaxis	Propranolol	GOV2 (54) IGV1 (10)	Cirrhosis	26 months (3–34)
Thakeb (1995)	Secondary prophylaxis	Ethanolamine	GOV1 (14) GOV2 (10) IGV1 (3)	Cirrhosis	1 year
Sarin (2002)	Secondary prophylaxis	Alcohol	GOV2 (28) IGV1 (9)	Cirrhosis and non‐cirrhotic	15.4 ± 3.7 months

GOV, gastroesophageal varices; GVL, gastric varices ligation; GVO, gastric varices obliteration; IGV, isolated gastric varices; n/a, not available; PTN, portal hypertension.

### Risk of bias

Two authors (Sakkarin Chirapongsathorn and Wuttiporn Manatsathit) independently performed the assessment of risk of bias of the included studies (Figures. S1 and S2). Only minor disagreements between the two reviewers were present and were resolved using discussion and consensus. Most included studies were considered to have low to medium risk of bias and unclear risk of bias. One study, published only as an abstract, was unassessable for bias.

### All‐cause mortality

**Figure 1:**
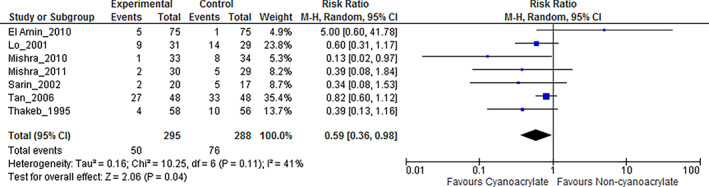
Comparison of mortality between the cyanoacrylate injection and non‐cyanoacrylate therapy. CI, confidence interval.

Meta‐analysis showed that the use of endoscopic cyanoacrylate injection was associated with significantly decreased risk of all‐cause mortality among patients with GV (RR, 0.59; 95% CI, 0.36–0.98, Fig. 1). However, the analysis may represent moderate heterogeneity without significance (I^2^ = 41%; *P* = 0.11). We did not identify evidence for publication bias qualitatively (Figure S3), or quantitatively using the Begg adjusted rank correlation test (*P* = 0.672). However, analysis of publication bias was not reliable because of the presence of heterogeneity and the small number of available studies.

### Subgroup analysis

**Figure 2:**
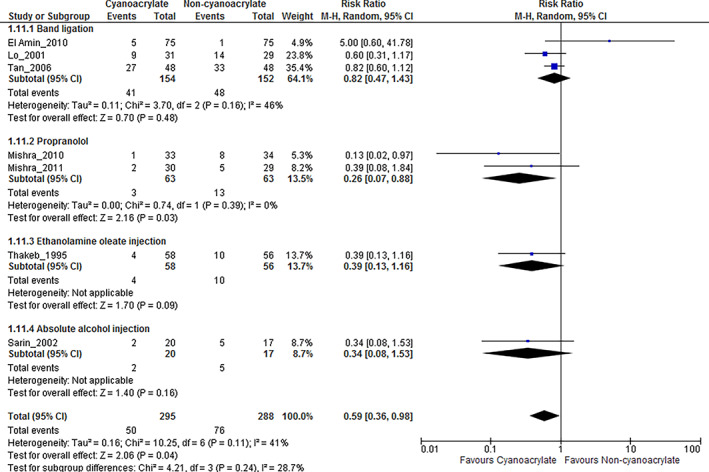
Mortality in each comparison treatment interventions. CI, confidence interval.

We performed preplanned subgroup analyses based on comparison among each treatment interventions (Fig. 2), and type of prophylactic care (Table 2). The use of endoscopic cyanoacrylate injection was associated with significantly decreased risk of all‐cause mortality when compared with the use of propranolol (RR, 0.26; 95% CI, 0.07–0.88; I^2^ = 0%). However, the use of endoscopic cyanoacrylate injection was unassociated with significantly decreased risk of all‐cause mortality when compared with other endoscopic interventions (band ligation; RR, 0.82; 95% CI, 0.47–1.43; I^2^ = 46%; ethanolamine oleate injection; RR, 0.39; 95% CI, 0.13–1.16; I^2^ was unapplicable; absolute alcohol injection; RR, 0.34; 95% CI, 0.08–1.53; I^2^ was not applicable). Heterogeneity was unapplicable among included single‐study comparisons.

**Table 2:** Subgroup and sensitivity analysis

						Tests of heterogeneity		
Subgroup analysis	No. of studies	No. of Death	Total no. of subjects	Relative risk	95% CI	*P*	I^2^ (%)	Heterogeneity between groups (*P* *)
Study or subgroup								
Peer‐reviewed articles	7	126	583	0.59	0.36–0.98	0.11	41	
Comparison interventions								
Band ligation	3	89	306	0.82	0.47–1.43	0.16	46	0.24
Propranolol	2	16	126	0.26	0.07–0.88	0.39	0	
Ethanolamine oleate	1	14	114	0.39	0.13–1.16	Not applicable	Not applicable	
Absolute alcohol	1	7	37	0.34	0.08–1.53	Not applicable	Not applicable	
Primary *versus* secondary prophylaxis								
Primary	1	7	59	0.39	0.08–1.84	Not applicable	Not applicable	0.59
Secondary	6	119	524	0.61	0.35–1.05	0.11	41	

* *P* ≤ 0.10, explains source of heterogeneity between groups.

### Sensitivity analysis

**Figure 3:**
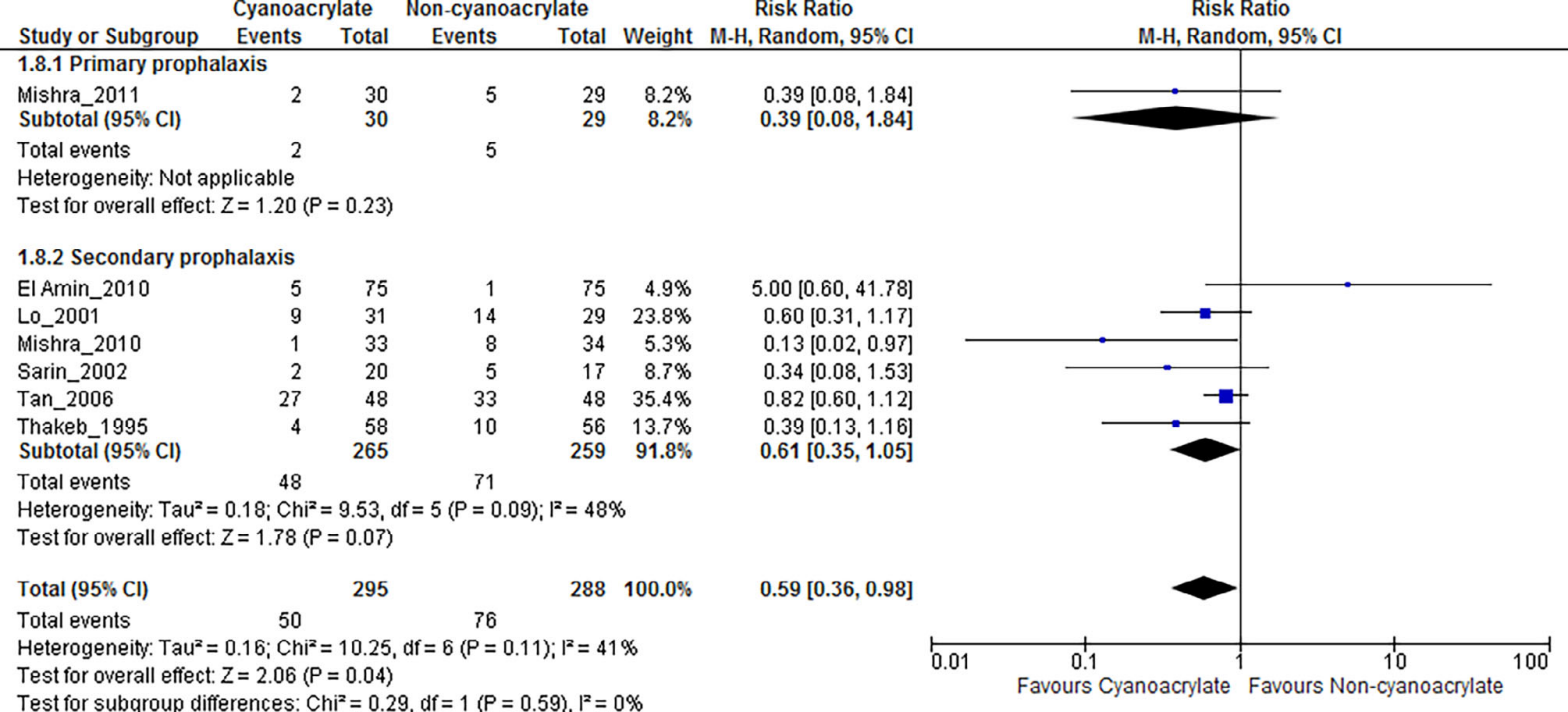
Mortality of cyanoacrylate therapy in primary and secondary prophylaxis. CI, confidence interval.

Only one study evaluated primary prophylaxis as the primary endpoint. The risk ratio of mortality rate of cyanoacrylate was not statically significant when compared with propranolol (RR, 0.39; 95% CI, 0.08–1.84; I^2^ was unapplicable, Fig. 3). Six studied using secondary prophylaxis as the secondary endpoint. Although a trend toward lower mortality rate was observed in the cyanoacrylate group compared with other treatment interventions, no statistical significance was found (RR, 0.61; 95% CI, 0.5–1.05; I^2^ = 41%, Fig. 3 and Table 2).

### Serious adverse event

**Figure 4:**
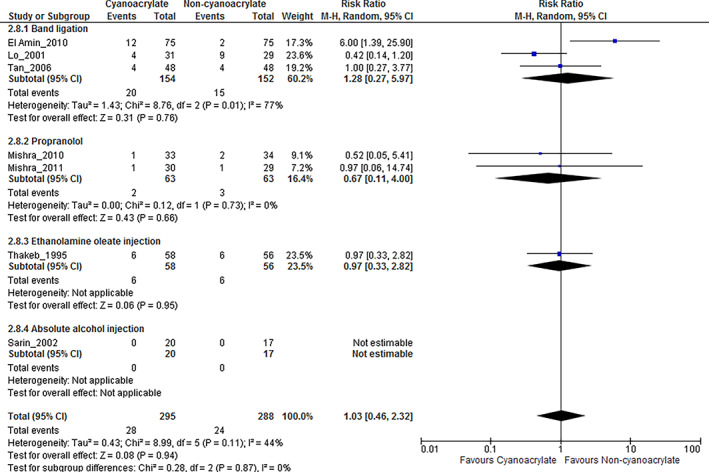
Serious adverse events in each comparison interventions. CI, confidence interval.

We defined a serious adverse event, as an adverse event involving any undesirable experience associated with the use of endoscopic cyanoacrylate injection and other comparison interventions including initial or prolonged hospitalization, disability or permanent damage, congenital anomaly or birth defect, required intervention to prevent permanent impairment or damage and other serious events. Overall, no statistical significance was found in adverse events of the cyanoacrylate and non‐cyanoacrylate groups (RR, 1.03; 95% CI, 0.46–2.32; I^2^ = 44%, Fig. 4). Also, no statistical significance was found when comparing cyanoacrylate with band ligation (RR, 1.28; 95% CI, 0.27–5.97; I^2^ = 77%), propranolol (RR, 0.67; 95% CI, 0.11–4; I^2^ = 77%), and ethanolamine (RR, 0.97; 95% CI, 0.33–2.28; I^2^ was unapplicable). Data regarding adverse events in the study comparing alcohol injection with cyanoacrylate were also unavailable.

### Bleeding rate after hemostasis

**Figure 5:**
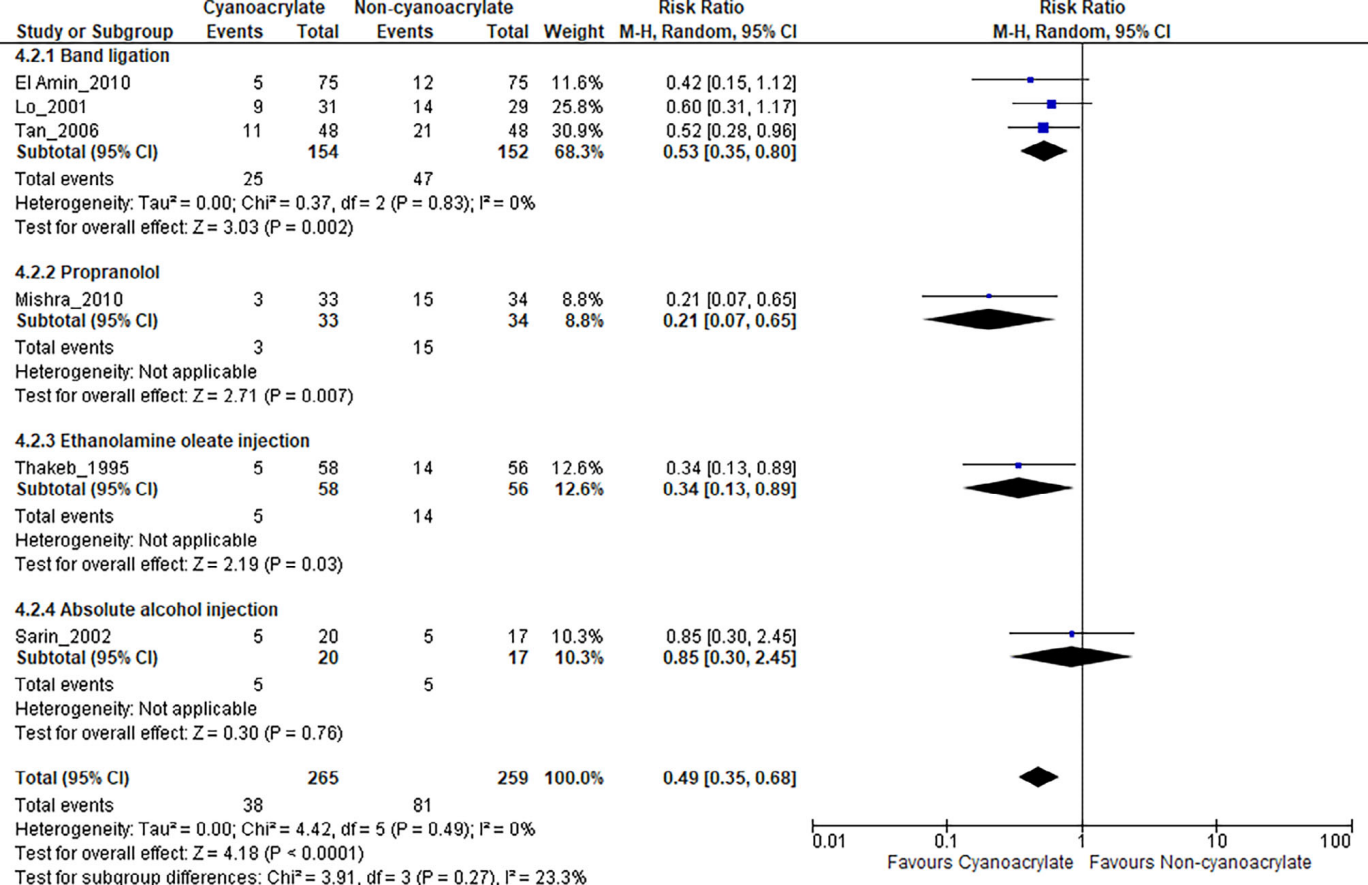
Bleeding after hemostasis in each comparison interventions. CI, confidence interval.

Risk ratio of bleeding events after hemostasis is shown in Figure 5. Overall, cyanoacrylate resulted in a significantly lower bleeding rate after hemostasis when compared with other treatment modalities (RR, 0.49; 95% CI, 0.35–0.68). A significant difference was found in bleeding rate after hemostasis when comparing the cyanoacrylate group with band ligation but not which alcohol injection (RR, 0.53; 95% CI, 0.35–0.80; I^2^ = 0% and R, 0.85; 95% CI, 0.13–2.45; I^2^ = unapplicable respectively). Bleeding after hemostasis did not apply to the study by Mishra 2011 as this study evaluated primary prophylaxis as the primary outcome.^14^ Predictably, endoscopic cyanoacrylate injection resulted in significantly less bleeding compared with propranolol (RR, 0.21; 95% CI, 0.07–0.65, I^2^ = unapplicable). When compared with ethanolamine injection, bleeding risk after hemostasis was significantly lower in the cyanoacrylate group (RR, 0.34; 95% CI, 0.13–0.89; I^2^ = unapplicable).

## Discussion

Our meta‐analysis demonstrated that overall cyanoacrylate injection resulted in lowered mortality rate compared with other treatment modalities for GV. Furthermore, cyanoacrylate also resulted in significantly lowered rate of bleeding after hemostasis compared with both propranolol, ethanolamine oleate injection, and band ligation. Additionally, our data demonstrated that cyanoacrylate appeared to be safe with similar adverse events compared with other treatment modalities.

Unlike EV, GV and its bleeding risk are not solely correlated with severity of portal hypertension or degree of hepatic venous portal gradient (HVPG). In fact, patients with GV tend to have lower HVPG compared with patients with EV and may occasionally present bleeding episodes despite HVPG values less than 12 mmHg.^20^ This has been postulated to be a result of more collateral venous circulation among patients with GV. As a result, propranolol has been proven ineffective in preventing GV bleeding episodes in a randomized placebo control trial.^21^ There were a few studies about bleeding GV prevention by using nonselective beta‐blocker (NSBB). However, there was a published RCT from Mishra *et al*.,^17^ which represented less efficacy of NSBB compared with cyanoacrylate injection in bleeding prevention but there was no difference in survival. Our study demonstrated that NSBB is less effective when compared with cyanoacrylate in both mortality and bleeding rates after hemostasis. This could confirm that NSBB alone should not be used and is inadequate to properly manage GV.

Although band ligation is still a recommended treatment for GOV1, several studies have suggested that its effectiveness is less than previously thought, and its role in managing GOV2 and IGV1 has been less studied. In this study, we found three high‐quality studies compared cyanoacrylate with band ligation. Our data suggested similar mortality rates but improved bleeding rates after hemostasis of cyanoacrylate compared with band ligation. Moreover, a study by El Amin clearly demonstrated that three sessions are required to achieve 99% hemostasis when using band ligation.^13^ In the study, the author also uniquely evaluated the efficacy of cyanoacrylate in the setting of active GV bleeding and demonstrated that cyanoacrylate is highly effective.^13^ Therefore, it appeared that cyanoacrylate results in better control of bleeding but without reduced mortality rate when compared with band ligation. However, it should be noted that most data were derived mostly from patients with GOV1 (240 patients) and a significantly smaller number of patients with GOV 2 and IGV1 (62 patients). Therefore, one can also argue that the similar efficacy of both band ligation and cyanoacrylate is mainly true for GOV1 but remains unclear for patients with GOV2 and IGV1. Another study aimed to compare, by using meta‐analysis, the effectiveness of cyanoacrylate injection *versus* band ligation for patients with acute GV bleeding.^22^ The result shows that compared with band ligation, cyanoacrylate injection has an advantage in the control of acute gastric variceal bleeding, also with lower recurrence rate and rebleeding (except GOV2).

However, this study includes non‐randomized control trial in analysis.^23^ Combination therapy between endoscopic ultrasound‐guided cyanoacrylate with coil embolization resulted in a better technical and clinical success compared with cyanoacrylate alone and coil embolization alone.^24^ However, using combination endoscopic therapy for GV is not commonly worldwide, and this is not the objective in our study.

Regarding efficacy of cyanoacrylate compared with ethanolamine oleate and alcohol, only two randomized controlled trials have been published. Our study demonstrated that trends have been observed toward significantly lower mortality rate for cyanoacrylate compared with ethanolamine oleate and alcohol. However, the conclusion cannot be drawn given the lack of adequate information. Theoretically, in an animal model, cyanoacrylate resulted in reduced variceal size. This could be explained by the high volume of blood flow through GV compared with EV, resulting in rapid flushing away of the sclerosing agent as compared with cyanoacrylate, which rapidly polymerizes and better obliterates varices.^25^ Further studies are required.

Regardless of the possible superior efficacy of cyanoacrylate, gastroenterologists have long been concerned about its adverse events, mainly emboli due to its rapid thrombogenicity. Several case reports have revealed embolic complications, which further raise this concern. Although our meta‐analysis and other related studies were not designed to address this point, sufficient data suggest higher morbidity rates and increased adverse events from cyanoacrylate.

Although we carefully selected only high‐quality studies to be included in this meta‐analysis, several instances of heterogeneity existed among the studies mainly involving the study methods resulting in a significant limitation of this study. Firstly, the characteristics of studied populations differed including type of GV and etiology of portal hypertension. Secondly, the main limitation of this study was the inadequate number of studies in each treatment group. The strongest data appeared to be in the propranolol and band ligation groups. Thirdly, using long‐term instead of short‐term mortality rates as the primary outcomes in this study may not have truly reflected the effect of managing GV given that the long‐term mortality rate depends more on the severity of underlying liver diseases.

In conclusion, despite significant limitations, we concluded that NSBB alone is inadequate to manage GV and should not be used. Overall, cyanoacrylate resulted in lower mortality rates compared with other treatment modalities combined. Additionally, cyanoacrylate clearly resulted in lowered bleeding rates after hemostasis and a trend toward lower mortality rate over band ligation was noted, but more studies are needed to confirm these results. Our study was unable to adequately compare cyanoacrylate with other sclerosing agents due to the lack of data for meaningful analysis. However, cyanoacrylate appeared to be safe with a similar rate of adverse events compared with other treatment modalities for GV.

## Supporting information

**Appendix S1.** Supporting information.

**Appendix S2.** Supporting information.

**Table S1.** Details of included studies.
**Table S2.** Objective and criteria of included studies.
