# Supplementary material for: Safety and efficacy of endoscopic cyanoacrylate injection in the management of gastric varices: A systematic review and meta‐analysis
Source: JGH Open. 2021 Jul 30;5(9):1047–55. doi: 10.1002/jgh3.12629 (PMC8454477; doi:10.1002/jgh3.12629)
Supplement: Supplementary file 1 — Appendix S1. Supporting information. [file JGH3-5-1047-s001.doc]

**Screening**

**Included**

**Eligibility**

**Identification**

1107 Records identified through database searching

5 Additional records identified through other sources

613 Records after duplicates removed

613 Records screened

580 Records excluded
Basic science

Reviews, case-reports, letters, editorials, commentaries

Not related to beta-blockers/cirrhosis

Death not reported

33 Full-text articles assessed for eligibility

23 Full-text articles excluded, Death not reported

Unable to calculated OR/RR

Studies from same population

Authors cannot provide ascites

Authors did not get back

No compare group

10 Studies included in qualitative synthesis

7 Studies included in quantitative synthesis (meta-analysis)

3 Full-text articles excluded, Absence of inclusion and exclusion criteria and mixed population of esophageal varices and gastric varices

**Supplementary Appendix C** Flowchart summarizing study identification and selection
