# Supplementary material for: Safety and efficacy of endoscopic cyanoacrylate injection in the management of gastric varices: A systematic review and meta‐analysis
Source: JGH Open. 2021 Jul 30;5(9):1047–55. doi: 10.1002/jgh3.12629 (PMC8454477; doi:10.1002/jgh3.12629)

Supplementary Appendix A

Keywords for Search Strategy

An experienced medical librarian developed a search strategy for “Safety and efficacy of cyanoacrylate injection in the management of patients with gastric variceal bleeding.” The strategy was run in the MEDLINE, Embase and Cochrane Database of Systematic Reviews, using the Ovid interface. The search was also run in the Web of Science and Scopus databases. There were no restrictions on publication date or language. Keywords and MeSH terms were used, including cyanoacrylate, dermabond and esophageal varices. The complete search strategies can be found in the appendix.

Databases searched on November 13, 2020:

- Ovid MEDLINE(R) 1946 to November Week 1 2020
  # of total results: 371
  after deduplication: 371
- Ovid Embase 1988 to 2020 Week 45
  # of total results: 449
  after deduplication: 240
- Ovid EBM Reviews - Cochrane Database of Systematic Reviews 2005 to September 2014
  # of total results: 2
  after deduplication: 2
- Scopus
- # of total results: 289
  after deduplication: 32
- Web of Science 1975-2020
  # of total results: 144
  after deduplication: 30

Database Search Strategies:

Database: Ovid MEDLINE(R) In-Process & Other Non-Indexed Citations and Ovid MEDLINE(R) <1946 to Present> Search Strategy:

--------------------------------------------------------------------------------

1 exp Cyanoacrylates/ (4041)

2 (cyanoacrylate*1 or N-butyl-2-cyanoacrylate or 2-octyl-cyanoacrylate or histoacryl or enbucrilate*1 or bucrylate*1

or "cyano acrylate" or cyanoacrylic or e910 or "e 910" or "eastman 910" or ernhaemone or alkylcyanoacrylate or dermabond or ocrilate or ocrylate or octylcyano* or "octyl2 cyanoacrylate").tw. (4170)

3 exp "Esophageal and Gastric Varices"/ (11580)

4 (((esophag* or oesophag*) adj3 (varice* or varicos* or varix*)) or ((esophag* or oesophag*) adj3 (hemorrhag* or

haemorrhag* or bleed*3))).tw. (8952)

5 (1 or 2) and (3 or 4) (389)

6 remove duplicates from 5 (371)

**Database: Embase <1988 to 2020 Week 45>**

Search Strategy:

--------------------------------------------------------------------------------

1 exp esophagus varices/ (11073)

2 (((esophag* or oesophag*) adj3 (varice* or varicos* or varix*)) or ((esophag* or oesophag*) adj3 (hemorrhag* or

bleed*3))).tw. (8434)

3 cyanoacrylate derivative/ or "poly(hexyl 2 cyanoacrylate)"/ or cyanoacrylate/ or "poly(isobutyl 2 cyanoacrylate)"/

or "poly(ethyl 2 cyanoacrylate)"/ (2993)

4 (cyanoacrylate*1 or 2-octyl-cyanoacrylate or histoacryl or enbucrilate*1 or bucrylate*1 or "cyano acrylate" or

cyanoacrylic or e910 or "e 910" or "eastman 910" or ernhaemone or alkylcyanoacrylate or dermabond or ocrilate or ocrylate or octylcyano* or "octyl2 cyanoacrylate" or N-butyl-2-cyanoacrylate).tw. (4231)

5 (1 or 2) and (3 or 4) (455)

6 remove duplicates from 5 (449)

**Database: EBM Reviews - Cochrane Database of Systematic Reviews <2005 to September 2020> Search Strategy:**

--------------------------------------------------------------------------------

1 (cyanoacrylate*1 or N-butyl-2-cyanoacrylate or 2-octyl-cyanoacrylate or histoacryl or enbucrilate*1 or bucrylate*1

or "cyano acrylate" or cyanoacrylic or e910 or "e 910" or "eastman 910" or ernhaemone or alkylcyanoacrylate or dermabond or ocrilate or ocrylate or octylcyano* or "octyl2 cyanoacrylate").tw. (13)

2 ((esophag* or oesophag*) adj2 (varice* or varicos* or varix or bleed* or hemorrhag* or haemorrhag*)).tw. (49)

3 1 and 2 (2)

**Scopus:**

( TITLE-ABS-KEY-AUTH ( ( esophag*  OR  oesophag* )  W/2  ( varice*  OR  varicos*  OR  varix  OR  bleed* OR  hemorrhag*  OR  haemorrhag* ) ) )  AND  ( TITLE-ABS-KEY-AUTH ( cyanoacrylate*1  OR  n-butyl-2-cyanoacrylate  OR  2-octyl-cyanoacrylate  OR  histoacryl  OR  enbucrilate*1  OR  bucrylate*1  OR  "cyano acrylate"  OR  cyanoacrylic  OR  e910  OR  "e 910"  OR  "eastman 910"  OR  ernhaemone  OR alkylcyanoacrylate  OR  dermabond  OR  ocrilate  OR  ocrylate  OR  octylcyano*  OR  "octyl2 cyanoacrylate" ) )

**Web of Science:**

| # 3 | [144](http://apps.webofknowledge.com/summary.do?product=WOS&doc=1&qid=5&SID=1EEbUOlV7MvuDWYQasQ&search_mode=CombineSearches&update_back2search_link_param=yes) | #2 AND #1  *Indexes=SCI-EXPANDED Timespan=All years* |
| --- | --- | --- |
| # 2 | [1,819](http://apps.webofknowledge.com/summary.do?product=WOS&doc=1&qid=2&SID=1EEbUOlV7MvuDWYQasQ&search_mode=AdvancedSearch&update_back2search_link_param=yes) | ts=(cyanoacrylate*1 or N-butyl-2-cyanoacrylate or 2-octyl-cyanoacrylate or histoacryl or enbucrilate*1 or bucrylate*1 or "cyano acrylate" or cyanoacrylic or e910 or "e 910" or "eastman 910" or ernhaemone or alkylcyanoacrylate or dermabond or ocrilate or ocrylate or octylcyano* or "octyl2 cyanoacrylate")  *Indexes=SCI-EXPANDED Timespan=All years* |
| # 1 | [7,654](http://apps.webofknowledge.com/summary.do?product=WOS&doc=1&qid=1&SID=1EEbUOlV7MvuDWYQasQ&search_mode=AdvancedSearch&update_back2search_link_param=yes) | ts=((esophag* or oesophag*) NEAR/2 (varice* or varicos* or varix or bleed* or hemorrhag* or haemorrhag*))  *Indexes=SCI-EXPANDED Timespan=All years* |

Supplementary Appendix B

Quality Assessment

The risk of bias and methodologic quality of the included studies was assessed using the Cochrane risk of bias tool for RCTs. The Cochrane risk of bias tool consisted of 7 domains as follow: (1) random sequence generation (selection bias), (2) allocation concealment (selection bias), (3) blinding of participants and personnel (performance bias), (4) blinding of outcome assessment (detection bias), (5) incomplete outcome data (attrition bias), (6) selective reporting (reporting bias), and (7) any other bias. Every domain could be classified as low risk of bias, high risk of bias, or as unclear risk of bias.

**Supplement Figure 1**. Risk of bias graph: review authors' judgements about each risk of bias item presented as percentages across all included studies.


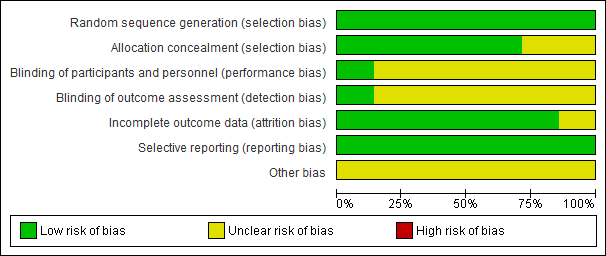


**Supplement Figure 2** Quality of included RCTs.


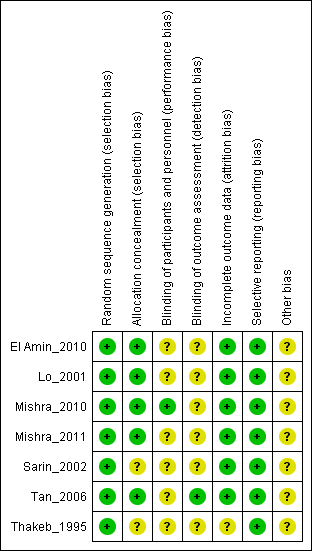


**Supplementary Figure 3** Publication bias assessment by funnel plot


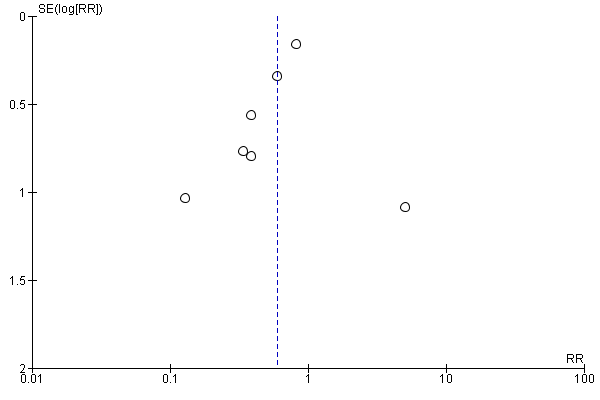

Supplement: Supplementary file 2 — Appendix S2. Supporting information. [file JGH3-5-1047-s003.docx]
