# Supplementary material for: Safety and efficacy of endoscopic cyanoacrylate injection in the management of gastric varices: A systematic review and meta‐analysis
Source: JGH Open. 2021 Jul 30;5(9):1047–55. doi: 10.1002/jgh3.12629 (PMC8454477; doi:10.1002/jgh3.12629)
Supplement: Supplementary file 3 — Table S1. Details of included studies. Table S2. Objective and criteria of included studies. [file JGH3-5-1047-s002.docx]

| **Supplementary table 1** Details of Included Studies  **Study** | **Duration of F/U** | **Cyanoacrylate Injection** | | | | | | **Control** | | | | | | |
| --- | --- | --- | --- | --- | --- | --- | --- | --- | --- | --- | --- | --- | --- | --- |
|  |  | **Mean/Median age, yr; sex, % males** | **No. of Childs A/B/C** | **Mean/Median MELD** | **Etiology of cirrhosis** | **Median HVPG** | **Type of Gastric varix** | **Control intervention** | **Mean/Median age, yr; sex, % males** | **No. of Childs A/B/C** | **Mean/Median MELD** | **Etiology of cirrhosis** | **Median HVPG** | **Type of Gastric varix** |
| El Amin et al, 2010 | 21 mo. | 51(4); 55(73.3) | 15/32/28 | Not reported | HBV, 14; HCV, 51; Bilharzial, 5; unknown, 5 | Not reported | GOV1, 75 | Band ligation | 52(6); 53(70.6) | 20/40/15 | Not reported | HBV, 14; HCV, 54; Bilharzial, 3; unknown, 4 | Not reported | GOV1, 75 |
| Mishra et al, 2011 | 28 mo. | 40 (10-71); 20 (66.7) | 10/12/8 | 13 (7-26) | ALD, 16; cryptogenic, 9; others, 5 | 14 (8-26) | GOV2, 26; IGV1, 4 | NSBB | 40 (10-65); 21 (72.4) | 9/11/19 | 13 (7-30) | ALD, 14; cryptogenic, 8; others, 7 | 14 (11-26) | GOV2, 25; IGV1, 4 |
| Lo et al, 2001 | 30 mo. | 58 (17); 62 (80.5) | 8/16/ 7 | Not reported | ALD, 10; HBV, 11; HCV, 7; HBV+HCV, 3 | Not reported | GOV1, 21; GOV2, 6; IGV, 4 | Band ligation | 55 (13); 22 (75.8) | 5/27/  7 | Not reported | ALD, 6; HBV, 11; HCV, 10; HBV+HCV, 2 | Not reported | GOV1, 20; GOV2, 8; IGV, 1 |
| Tan et al, 2006 | 60 mo. | 61 (14.6), 35 (71.4) | 13/26/ 10 | Not reported | Viral, 31; ALD, 3; mixed, 4; others, 12 | Not reported | GOV1, 27; GOV2, 9; IGV1, 13 | Band ligation | 61(12.3); 34(70.8) | 12/25/  11 | Not reported | Viral, 32; ALD, 3; mixed, 4; others, 9 | not reported | GOV1, 26; GOV2, 16; IGV1, 6 |
| Mishra et al, 2010 | 32 mo. | 40 (6-70), 19 (57.5) | 4/12/17 | 15(8-35) | ALD,12; crytogenic, 14; viral+others 7 | 15 (10-23) | GOV2, 26; IGV1, 6 | NSBB | 39 (6-70), 26 (76.4) | 5/13/16 | 15(8-35) | ALD,11; crytogenic, 15; viral+others 8 | 14 (11-24) | GOV2, 28; IGV1, 4 |
| Thakeb et al, 1995*^a,b^* | 24 mo. | 44 (24-70), 46 (79.3) | 16/33/9 | Not reported | Viral, 8; Schistosomaiasis, 38; others, 12 | Not reported | GOV, 17  ; IGV, 2*^b^* | Ethanolamine oleate 5%*^a^* | 43 (20-70), 49 (87.5) | 9/38/9 | Not reported | Viral, 8; Schistosomaiasis, 37; others, 11 | Not reported | GOV, 7  ; IGV, 1*^b^* |
| Sarin et al, 2002 | 36 mo. | 36.1 (12.2), 15 (75) | 9/9/2 | Not reported | Cirrhosis, 11; NCPF, 4; EHPVO, 5 | Not reported | IGV1, 20 | Absolute alcohol | 34.5 (12.9), 12 (70.5) | 8/6/3 | Not reported | Cirrhosis, 7; NCPF, 4; EHPVO, 6 | Not reported | IGV1, 17 |

NOTE. Data are expressed as means (SD), median (IQR) or as absolute (percentage).

ALD, alcoholic liver disease; EHPVO = extrahepatic portal vein obstruction; HCV, hepatitis C; HBV, hepatitis B; GOV, gastroesophageal varices; HVPG, hepatic venous pressure gradient; IGV, isolated gastric varices; NCPF = non-cirrhotic portal fibrosis; NSBB, non-selective betablocker viral, viral hepatitis; TIPS, transjugular intrahepatic portosystemic shunt; Childs, Child-Pugh score

*^a^*The study of Thakeb et al reported the combined Histoacryl-*N­*-butyl-2-cyanoacrylate and ethanolamine oleate 5% compared to ethanolamine oleate 5% alone.

*^b^*The study of Thakeb et al reported patient characteristics in the overall type of GOV and IGV, did not classified as type 1 or 2.

| **Study**  **Supplementary table 2** Objective and Criteria of Included Studies | **Study country** | **Time period** | **Setting** | **Objective** | **Inclusion criteria** | **Exclusion criteria** |
| --- | --- | --- | --- | --- | --- | --- |
| El Amin et al | Egypt | January 2008-September 2009 | Secondary prophylaxis | To compare the efficacy and safety of endoscopic variceal ligation versus endoscopic cyanoacrylate injection in the treatment of bleeding junctional varices | Criteria included: (1) Patients with portal hypertension based on results of clinical, laboratory and imaging studies; (2) Patients with clinical signs of hematemesis, melena or hematochezia; (3) Patients with active bleeding from junctional vaices which was defined as active spurting or oozing of blood from the junctional varix; (4) Patients who gave informed written consent. | Criteria were: (1) Undetermined source of bleeding; (2) Previous history of sclerotherapy, band ligation or shunt operation; (3) Patients with hepatic encephalopathy or hepatorenal syndrome; (4) Associated advanced HCC, cerebral vascular accidents or other debilitating diseases; (5) Non-compliant patients or patients unwilling to sign an informed consent. |
| Mishra et al | India | August 2006-June 2009 | Primary prophylaxis | To compare the efficacy of endoscopic cyanoacrylate injection versus betablockers, versus no treatment in the prevention of GV bleeding | Cirrhotic patient with GOV2 (eradicated EV) or IGV1, who had never bled from GV, were included. | Patients with presence of EV, non-cirrhotic portal hypertension, acute bleed or past history of bleed from GV, contraindications to beta-blockers and cyanoacrylate injection, prior treatment for prevention of bleeding from GV, patients on beta-blockers, hepatic encephalopathy grade III/IV, hepatorenal syndrome, hepatocellular carcinoma, presence of deep jaundice (serum bilirubin >10 mg/dl), cardiorespiratory failure, age >75 years, pregnancy, and patients not giving informed consent |
| Lo et al | Taiwan | July 1996-December 1999 | Secondary prophylaxis | To compare the efficacy and safety of endoscopic obturation using butyl cyanoacrylate and band ligation in the management of gastric variceal bleeding | Patients with cirrhosis between 20 and 70 years of age with the history of gastric variceal bleeding admitted to Kaohsiung Veterans General Hospital from July 1996-December 1999 | Criteria were (1) undetermined origin of bleeding from esophageal varices or gastric varices; (2) presence of deep jaundice (serum bilirubin >10 mg/dL), hepatic encephalopathy, or hepatorenal syndrome; (3) association with advanced hepatocellular carcinoma, Okuda-staging III, uremia, cerebral vascular accident, or other debilitating disease; (4) prior history of sclerotherapy or shunt operation; and (5) life expectancy less than 24 hours. Betablockers were not administered during the study. |
| Tan et al | Taiwan | July 1996-June 2002 | Secondary prophylaxis | To compare the efficacy of cyanoacrylate injection and band ligation in the treatment of acute GVH in liver patients with cirrhosis with or without concomitant HCC | Patients who were aged between 18 and 80 years and had endoscopy-proven acute GVH were included. GVH was diagnosed using the following: (1) clinical signs of hematemesis, coffee ground vomitus, hematochezia, or melena; (2) endoscopic signs of an active spurting or oozing from the GOV; (3) adherent blood clots, white nipple signs, or erosions on the GOV; or (4) in the presence of distinct large GOV with a red-color sign and absence of EV and other bleeding sources. | Criteria were: (1) had previous endoscopic, surgical treatment or transjugular intrahepatic portal systemic shunt for GVH; (2) had a terminal illness of any major organ system, such as heart failure, uremia, chronic obstructive pulmonary disease, or nonhepatic malignancy. |
| Mishra et al | India | August 2006-March 2009 | Secondary prophylaxis | To study the efficacy of endoscopic cyanoacrylate injection versus betablockers in the prevention of gastric variceal (GOV2 or IGV1) rebleeding and improvement in survival | Patients who had bled from GOV2 or IGV1 were included. | Patients with non-cirrhotic portal hypertension, presence of oesophageal varices, contraindications to betablockers and cyanoacrylate injection, patients already on betablockers, past history of cyanoacrylate injection or sclerotherapy for GOVs or GOV ligation, TIPS, BRTO, balloon-occluded endoscopic injection sclerotherapy, shunt operation, undetermined origin of bleeding, hepatic encephalopathy grade III/IV, hepatorenal syndrome, HCC, presence of deep jaundice (serum bilirubin >10 mg/dl), cardiorespiratory failure, age >75 years, pregnancy or patients who did not give informed consent. |
| Thakeb et al | Egypt | April 1990-April 1992 | Secondary prophylaxis | To compare the combined use of *N*-butyl-2-cyanoacrylate and ethanolamine oleate with ethanolamine oleate alone | Patients admitted to the Gastrointestinal Endoscopy Unit, Kasr El Aini Hospital from April 1990-April 1992 with the following characteristics: (1) recent (within 48 hours) and documented variceal bleeding, (2) stable hemodynamic conditions (i.e., after resuscitation from shock) | Patients with major associated medical problems, such as recent ischemic heart disease (i.e., myocardial infarction or unstable angina), advanced lung disease, or the presence of multiple neoplastic hepatic lesions |
| Sarin et al | India | 1995-1998 | Secondary prophylaxis | To evaluate the relative efficacy and safety of gastric variceal sclerotherapy, using alcohol as a sclerosant, and gastric variceal obturation, using cyanoacrylate glue, in the management of patients who were actively bleeding or had bled from IGVs. | Patients with portal hypertension,  and having IGVs with histories of variceal bleeding. | Criteria were (1) Patients who were undergoing endoscopic sclerotherapy/ banding for esophageal varices, (2) patients with hepatorenal syndrome or higher than grade II hepatic encephalopathy, and (3) patients not giving informed consent for endoscopic procedures |

BRTO, balloon-occluded retrograde transvenous obliteration; EV, esophageal varices; GV or GOV, gastroesophageal varices; GVH, gastric variceal hemorrhage; HCC, hepatocellular carcinoma; IGV, isolated gastric varices; TIPS, transjugular intrahepatic portosystemic shunt
